# Novel therapies for multiple myeloma

**DOI:** 10.18632/aging.101284

**Published:** 2017-08-28

**Authors:** Craig T. Wallington-Beddoe, Stuart M. Pitson

**Affiliations:** Centre for Cancer Biology, University of South Australia and SA Pathology, Adelaide, Australia

**Keywords:** myeloma, therapy, proteasome inhibitor, immunomodulatory agent, monoclonal antibody

Multiple myeloma (MM) is an incurable blood cancer of plasma cells that occurs in older individuals with a median age at diagnosis of 69 years and a median overall survival of 6-7 years [1, 2]. Over the past two decades there has been an explosion of novel agents that have dramatically improved overall response rates (ORR), progression-free survival (PFS) and overall survival (OS). Accordingly, the median OS for 2001-2005 was 4.6 years, increasing to 6.1 years for 2006-2010 [1]. The main classes of novel agents include proteasome inhibitors, immunomodulatory agents and monoclonal antibodies. These agents are typically used in doublet or triplet regimens that include a chemotherapeutic drug and/or corticosteroid, although regimens are emerging that are chemotherapy-free.

Proteasome inhibitors are potent anti-MM agents which inhibit one or more proteolytic subunits of the 26S proteasome. Their efficacy is generally related to the degradation of pro-survival factors including NF-κB signalling, induction of endoplasmic reticulum stress and modification of the bone marrow micro-environement rendering it less supportive of MM cell growth [3]. The first-in-class reversible 26S proteasome inhibitor Bortezomib received FDA approval in 2003 and is commonly used in combination with a chemotherapeutic agent and corticosteroid resulting in an ORR over 80% in newly-diagnosed MM patients that generally translates into improvements in PFS and OS [4]. The second generation, irreversible 26S proteasome inhibitor Carfilzomib is able to re-sensitize 24% of Bortezomib-refractory MM patients and when combined with Dexamethasone in the relapsed/refractory setting results in an ORR of 77% and median PFS of 18.7 months, compared to 63% and 9.4 months, respectively, in patients treated with Bortezomib [5]. Ixazomib, the first orally available proteasome inhibitor, produces an ORR of 15-20% alone and 30-50% when combined with Dexamethasone in relapsed/refractory MM patients [6]. However, an ORR of 78% and median PFS of 20.6 months were achieved when Ixazomib was combined with Lenalidomide and Dexamethasone compared to 72% and 14.7 months, respectively, without Ixazomib [6]. No direct comparisons have yet been made between Ixazomib and Bortezomib, although data to date suggests at least equal efficacy with the advantage of being an oral agent.

The immunomodulatory drugs (IMiDs), Thalidomide, Lenalidomide and Pomalidomide have also made a major impact in the management of MM. The anti-MM effects of IMiDs are related to their binding to the ubiquitin ligase cereblon (CRBN) and subsequent ubiquitination and degradation of two B-cell transcription factors, Ikaros (IKZF1) and Aiolos (IKZF3) [7]. Thalidomide was the first-in-class IMiD, FDA-approved for newly-diagnosed MM patients in 2006. Despite a checkered history in the 1950s and 1960s due to teratogenicity, it has high anti-MM activity and has been incorporated into many treatment regimens. In 2006, the second generation IMiD, Lenalidomide, was FDA-approved for relapsed/refactory MM patients and more recently for use in newly-diagnosed patients in combination with Dexamethasone, with ORR 40-60% in the former group of patients and 68-91% in the latter [7]. Furthermore, continuous Lenalidomide and Dexamethasone in newly diagnosed patients increased PFS (26.0 vs 21.0 months) and doubled the 4-yr OS (33% vs 14%) compared to a fixed duration of therapy [7]. Several phase III studies have compared Lenalidomide and Dexamethasone with the combination of Lenalidomide, Dexamethasone and a proteasome inhibitor or monoclonal antibody resulting in further improvements in ORR, PFS and OS [7]. Pomalidomide is a third generation IMiD approved for relapsed/refractory MM patients who have previously been treated with Bortezomib and Lenalidomide. In combination with Dexamethasone, the ORR was found to be 33% with PFS 4.6 months and OS 11.9 months [7].

Monoclonal antibodies show promise of further improvements in response rates when added to other MM drug classes. The anti-CD38 monoclonal antibody Daratumumab produced an ORR of approximately 30% when administered as a single agent to relapsed/refractory MM patients which increased to 83% (12-month PFS 61%) and 93% (12-month PFS 83%) when combined with Bortezomib or Lenalidomide and Dexamethasone, respectively [8]. Elotuzumab binds to signalling lymphocytic activation molecule family member 7 (SLAM7) reducing MM cell binding to bone marrow stroma and activating antibody-dependent cell-mediated cytotoxicity [8]. Interestingly, whilst no responses to Elotuzumab as a single agent were observed, the addition of Elotuzumab to Lenalidomide and Dexamethasone in relapsed/refactory MM patients resulted in an ORR of 79% versus 66% without Elotuzumab which translated into improved PFS [8]. Lastly, Pembrolizumab targets the programmed death 1 (PD-1)/programmed cell death ligand 1 (PD-L1) pathway, a critical initiator of immune activation, playing a role in mediating tolerance [8]. When combined with Lenalidomide and Dexa-methasone in relapsed/refractory MM patients, Pembrolizumab results in an ORR of 76% [8].

Currently, the use of high-dose therapy (HDT) and autologous stem cell transplantation remains the standard of care for physically fit newly-diagnosed MM patients [2]. However, with the continuing development of novel agents, chemotherapy-free regimens that combine a monoclonal antibody with two other novel agents will likely obviate the need for HDT in this setting. It is also worth mentioning that early clinical trials investigating chimeric antigen receptor T-cells (CAR T-cells) that target MM are encouraging with this approach likely to gain more attention in future. Thus, the armoury of novel agents for treating MM is rapidly growing and the challenge will be to conduct well-designed clinical trials to determine the optimal combination of these agents in various clinical settings. Of course, novel agents are expensive and convincing governments to agree to fund these combinational therapies remains the plight of the physician.
